# Association of Oral Health Literacy and Oral Health Knowledge with Risk Behaviors and Self-Rated Oral Health Among Chinese Dental Patients: A Hospital-Based Cross-Sectional Study in Guangzhou

**DOI:** 10.3390/healthcare14203444

**Published:** 2026-10-14

**Authors:** Jiali Yu, Zhe Ji, Rui Jiang, Rafiqul Islam, Xiang Li, Ermin Nie

**Affiliations:** 1 First Affiliated Hospital, Sun Yat-Sen University, 58 Zhong Shan Road 2nd, Guangzhou 510080, China; yujli7@mail.sysu.edu.cn (J.Y.); jizh7@mail.sysu.edu.cn (Z.J.); jiangr9@mail.sysu.edu.cn (R.J.); hpyylx@163.com (X.L.); 2 Department of Restorative Dentistry, Faculty of Dental Medicine, Hokkaido University, Kita 13, Nishi 7, Kita-ku, Sapporo 060-8586, Japan; rony12cdc@den.hokudai.ac.jp

**Keywords:** oral health literacy, oral health knowledge, risk behaviors, preventive oral-health practices, self-rated oral health, oral health promotion

## Abstract

**Background:** This study investigated the associations of oral health literacy and oral health knowledge with oral health-related behaviors and self-rated oral health among Chinese dental patients attending a tertiary hospital in Guangzhou, China. **Methods:** A hospital-based cross-sectional study was conducted among 323 adult dental patients from 1 December 2025 to 28 February 2026. Data were collected using a structured self-administered questionnaire assessing sociodemographic characteristics, oral health literacy, oral health knowledge, risk behaviors, preventive oral-health practices, and self-rated oral health. Descriptive statistics, Cronbach’s alpha analysis, Spearman’s rank correlation, multiple linear regression, and ordinal logistic regression analyses were performed. **Results:** Participants demonstrated relatively high levels of oral health literacy and oral health knowledge. Oral health literacy was positively correlated with oral health knowledge (ρ = 0.602, *p* < 0.001) and negatively correlated with risk behaviors (ρ = −0.238, *p* < 0.001). Ordinal logistic regression analysis demonstrated that higher risk behavior scores were significantly associated with lower odds of reporting better self-rated oral health (Estimate = −0.104, *p* < 0.001). Multiple linear regression analysis showed that age (β = 0.180, *p* = 0.001), oral health literacy (β = −0.355, *p* < 0.001), and oral health knowledge (β = 0.199, *p* = 0.002) were significantly associated with risk behavior scores. Higher risk behavior scores were independently associated with poorer self-rated oral health (β = −0.214, *p* < 0.001). **Conclusions:** Chinese dental patients demonstrated satisfactory levels of oral health literacy and oral health knowledge. Higher oral health literacy was associated with fewer oral health-related risk behaviors, while greater engagement in risk behaviors was associated with poorer self-rated oral health.

## 1. Introduction

Oral diseases remain among the most prevalent non-communicable diseases worldwide, affecting approximately 3.5 billion people and substantially impairing quality of life through pain, tooth loss, impaired mastication, and reduced social well-being [1,2,3]. Dental caries, periodontal diseases, and tooth loss continue to impose a considerable public health burden despite advances in preventive care [1,2,4]. Oral diseases are influenced by common lifestyle factors such as sugar intake, tobacco use, and alcohol consumption. Therefore, promoting healthy behaviors and preventive strategies is important for improving oral health worldwide [1,2].

Oral health literacy (OHL) refers to an individual’s ability to obtain, understand, evaluate, and use oral health information to make appropriate decisions regarding oral disease prevention and management [5,6]. Individuals with higher OHL are generally better able to understand and use oral health information, which may help them adopt healthier behaviors and seek preventive dental care. Conversely, limited OHL may be associated with inadequate self-care and delayed treatment seeking [7,8]. However, previous studies have reported inconsistent findings regarding the strength and pathways of these relationships across different populations [7,8,9].

OHL and oral health knowledge are closely related; however, they represent distinct constructs. Oral health knowledge refers to factual understanding of oral diseases, preventive measures, and oral health practices, whereas OHL encompasses broader competencies, including the ability to obtain, understand, evaluate, and apply oral health information in health-related decision-making [8]. Individuals may possess adequate oral health knowledge but still experience difficulties interpreting and using oral health information in daily life. Therefore, examining OHL and oral health knowledge simultaneously may provide a more comprehensive understanding of their relationships with oral health-related behaviors and oral health outcomes.

China has experienced considerable improvements in oral healthcare services and access to dental care during recent decades [10]. Nevertheless, oral diseases continue to represent an important public health burden, while preventive oral health behaviors remain suboptimal among many population groups [10,11]. Previous studies conducted in China have identified deficiencies in oral health knowledge, attitudes, and preventive practices among both patients and healthcare professionals [12,13,14]. Oral health knowledge and preventive behaviors have been shown to vary according to age, educational level, and socioeconomic status [12,13]. Given China’s large population and increasing need for dental care, it is important to understand the factors that influence oral health behaviors and oral health outcomes. This information can help develop more effective oral health promotion and prevention programs [10,11].

Dental patients attending tertiary-care hospitals represent an important clinical population because they actively utilize oral healthcare services and frequently receive oral health information, education, and preventive recommendations from dental professionals. Understanding the relationships among OHL, oral health knowledge, behaviors, and self-rated oral health within this setting may help inform the development of more effective patient-centered oral health promotion strategies. However, findings from tertiary-care patients may not necessarily be generalizable to broader community populations.

Despite the growing body of research on OHL, important knowledge gaps remain. Most previous studies in China have focused on community populations, older adults, students, or healthcare professionals rather than patients actively seeking dental care [12,13,14,15]. Furthermore, relatively few studies have simultaneously examined OHL, oral health knowledge, oral health-related behaviors, and self-rated oral health within a single analytical framework [13,14,15]. As a result, evidence remains limited regarding how these interrelated factors are associated among Chinese dental patients in clinical settings. Addressing this gap is important because dental patients regularly receive oral health information and preventive advice from healthcare professionals, making them a relevant population for understanding the role of literacy- and knowledge-related factors in oral health behaviors and outcomes.

Therefore, this study aimed to evaluate OHL, oral health knowledge, risk behaviors, preventive oral-health practices, and self-rated oral health among Chinese dental patients attending a tertiary hospital and to investigate the associations among these factors and selected sociodemographic characteristics. By simultaneously examining these factors in a clinical population, the study seeks to provide evidence that may inform behavior-focused oral health promotion strategies. Based on the previous literature, we hypothesized that (1) higher OHL would be associated with greater oral health knowledge, (2) higher OHL and oral health knowledge would be associated with fewer oral health-related risk behaviors, and (3) lower levels of risk behaviors would be associated with better self-rated oral health.

## 2. Materials and Methods

### 2.1. Study Design and Setting

This hospital-based cross-sectional study was conducted among dental patients attending the First Affiliated Hospital of Sun Yat-sen University, Guangzhou, China. Data were collected over a three-month period from 1 December 2025 to 28 February 2026.

The study was designed and reported in accordance with the Strengthening the Reporting of Observational Studies in Epidemiology (STROBE) guidelines for cross-sectional studies. Ethical approval was obtained from the Institutional Ethics Committee (IEC) for Clinical Research and Animal Trials of the First Affiliated Hospital of Sun Yat-sen University (Approval No. 2025/573; Date: 15 August 2025). All participants provided written informed consent before enrolment. Participation was voluntary, and all information was collected anonymously to ensure confidentiality.

### 2.2. Sample Size Calculation

The sample size for this cross-sectional study was calculated using the single-proportion formula:

$$n=\frac{Z^{2}p\left(1-p\right)}{d^{2}}$$

Assuming a prevalence of OHL 70% based on findings from our preliminary pilot study, with a confidence interval 95% (Z = 1.96) and a margin of error 5% (d = 0.05) were applied, resulting in a required sample size of 323. To account for potential incomplete questionnaires or missing data, an additional 20 participants were planned, resulting in a target of 343 participants to be invited. During the recruitment process, 343 patients were invited to participate. Among the initial 323 participants, 7 questionnaires were found to be incomplete. These 7 participants were replaced by 7 participants from the additional 20 participants. The remaining 13 additional participants were not included in the final analysis. Therefore, the final analytical sample consisted of 323 participants with complete questionnaires and were included in the final analysis, corresponding to a completion rate of 94.2% (323/343). The participant recruitment and selection process is shown in Figure S1. Although the initial sample size calculation was based on a prevalence-oriented approach, the final sample size was also considered in relation to the number of predictors included in the regression models. Based on Green’s rule of thumb (N ≥ 50 + 8k), where k represents the number of predictors, a minimum of 130 participants would be required for a model with 10 predictors and 138 participants for a model with 11 predictors. The final sample of 323 participants therefore exceeded these rule-of-thumb thresholds for the planned multivariable regression analyses.

### 2.3. Participants and Sampling

During the study period, adult dental patients attending the outpatient dental clinics of the First Affiliated Hospital of Sun Yat-sen University were invited to participate in the survey. Eligible patients were consecutively invited to participate during routine outpatient visits throughout the study period using a convenience sampling approach. Inclusion criteria included being aged 18 years or older, able to read and understand questionnaire, attending the dental clinic during the study period, and willing to provide written informed consent. Patients with serious medical or cognitive conditions that could impair their ability to complete the questionnaire, and questionnaires containing substantial missing data were excluded from the study. A total of 323 participants completed the questionnaire and met the eligibility criteria, and their responses were included in the final analysis.

### 2.4. Questionnaire Structure and Content

The questionnaire consisted of six sections. The complete questionnaire used in this study is provided as Supplementary File S1.

#### 2.4.1. Sociodemographic Characteristics

The questionnaire collected information on participants’ sociodemographic characteristics, including age, sex, educational attainment, and monthly income. Monthly income was recorded in Renminbi (RMB), the official currency of China, and categorized into four levels: <3000 RMB (lower income), 3000–5000 RMB (lower-middle income), 5000–8000 RMB (upper-middle income), and >8000 RMB (higher income).

#### 2.4.2. OHL

OHL was assessed using eight items designed to evaluate participants’ ability to understand oral health information provided by dentists, comprehend written oral health materials, identify reliable sources of oral health information, assess the trustworthiness of oral health information, follow dental instructions, communicate effectively with dental professionals, understand the importance of preventive oral care, and use oral health information to make informed decisions regarding their oral health. The OHL items were adapted from previously OHL instruments and studies [16,17,18]. Responses to each item were measured using a five-point Likert scale ranging from 1 (strongly disagree) to 5 (strongly agree). The total OHL score was calculated by summing the item scores, with higher scores indicating greater OHL. Item scores were summed to obtain a total OHL score ranging from 8 to 40, with higher scores indicating greater OHL.

#### 2.4.3. Oral Health Knowledge

Oral health knowledge was assessed using four items that evaluated participants’ knowledge regarding the preventive effect of fluoride on dental caries, the significance of bleeding gums as an indication of oral disease, the relationship between sugary food consumption and dental caries, and the recommendation to brush teeth at least twice daily. The oral health knowledge items were adapted from previous studies assessing knowledge of oral disease prevention and oral health practices among Chinese populations [12,14]. Each item was scored as either correct or incorrect, with correct responses assigned a score of 1 and incorrect responses assigned a score of 0. Item scores were summed to obtain a total knowledge score ranging from 0 to 4, with higher scores indicating greater oral health knowledge.

#### 2.4.4. Risk Behaviors

Risk behaviors were evaluated using seven items related to sweet food consumption, sugary beverage consumption, snacking before bedtime, smoking, alcohol consumption, skipping toothbrushing at night, and delaying dental treatment. Participants reported the frequency of these behaviors using response categories such as never, sometimes/weekly, and daily. The risk behavior and preventive oral-health practice items were adapted from previously published studies assessing oral health-related behaviors among adult dental patients [12,19]. For the seven risk behavior items, responses were scored as 0 = never, 1 = sometimes/weekly, and 2 = daily. The item scores were summed to generate a total risk behavior score ranging from 0 to 14, with higher scores indicating greater engagement in oral health-related risk behaviors.

#### 2.4.5. Preventive Oral-Health Practices Index

Preventive oral-health practices were assessed using seven items that evaluated participants’ oral hygiene and preventive dental care behaviors. The items included toothbrushing frequency, use of fluoride toothpaste, regular use of dental floss, regular use of mouthwash, attendance at regular dental check-ups, receipt of professional dental cleaning during the previous year, and preventive dental visits. The preventive oral-health practice items were adapted from previous studies evaluating preventive oral health behaviors among adults [12,19]. For each preventive oral-health practice item, participants received a score of 1 if they reported the recommended behavior and 0 if they did not. The item scores were summed to create a preventive oral-health practice index ranging from 0 to 7, with higher scores indicating greater engagement in recommended preventive oral-health practices.

#### 2.4.6. Self-Rated Oral Health

Self-rated oral health was assessed using a single global item in which participants rated their overall oral health. This variable was used as the primary oral health outcome. In addition, three related oral health indicators were collected descriptively, including dental pain during the previous 12 months, gingival bleeding during toothbrushing, and overall satisfaction with oral health. These variables were included to provide supplementary information regarding participants perceived oral health condition but were not combined into a composite score and were not used as outcome variables in the regression analyses. Self-rated oral health items were adapted from previous study examining self-rated oral health and oral health-related outcomes [20]. Self-rated oral health was coded on a five-category ordinal scale ranging from 1 (poor) to 5 (excellent), with higher scores indicating better perceived oral health status.

Composite scores were calculated for the OHL, oral health knowledge, risk behavior, and preventive oral-health practice domains. Higher scores represented higher OHL, greater oral health knowledge, greater engagement in oral health-related risk behaviors, and more favorable preventive oral-health practices, respectively.

### 2.5. Questionnaire Development and Validation

Questionnaire items were adapted from previously published studies and instruments assessing OHL, oral health knowledge, oral health-related behaviors, preventive oral-health practices, and self-rated oral health [16,17,18,19,20]. The original questionnaire was prepared in English and translated into Chinese by bilingual researchers experienced in oral health research. To ensure conceptual and linguistic equivalence, the translated version was independently reviewed and compared with the original English version. The translated questionnaire was independently reviewed by two experienced dental experts to evaluate the clarity, relevance, and appropriateness of the items. Based on their recommendations, minor wording modifications were made to improve readability and comprehensibility.

Prior to the main survey, a pilot study was conducted among 30 dental patients who met the study eligibility criteria. Participants were asked to complete the questionnaire and provide feedback regarding item clarity, wording, response options, and completion time. No substantial difficulties were reported, and minor revisions were made to improve question wording and formatting. Data obtained during the pilot phase were not included in the final analysis. The reliability of the questionnaire was evaluated using Cronbach’s alpha coefficient.

### 2.6. Reliability Assessment

The internal consistency reliability of the questionnaire domains was evaluated using Cronbach’s alpha coefficient. Cronbach’s alpha values of 0.70 or higher were considered indicative of acceptable internal consistency. Reliability analyses were performed for the OHL, oral health knowledge and risk behavior domains. For descriptive purposes, Cronbach’s alpha was also calculated for the preventive oral-health practice items. However, this measure should be interpreted cautiously because the items represent distinct preventive behaviors rather than indicators of a single latent construct.

### 2.7. Statistical Analysis

Data were analyzed using IBM SPSS Statistics version 29.0 (IBM Corp., Armonk, NY, USA). Descriptive statistics were used to summarize participant characteristics and questionnaire responses. Continuous variables were presented as means and standard deviations (SDs), whereas categorical variables were expressed as frequencies and percentages. Internal consistency was assessed using Cronbach’s alpha coefficients. Spearman’s rank correlation analysis was performed to examine associations among age, OHL, oral health knowledge, risk behaviors, and preventive oral health practices because several variables were ordinal or not normally distributed. Multiple linear regression analyses were conducted to identify factors associated with risk behavior scores and self-rated oral health status. Independent variables entered into the regression models included age, sex, educational level, monthly income, OHL, oral health knowledge, and risk behavior score (for the self-rated oral health model). Because self-rated oral health was an ordinal outcome with five ordered categories, ordinal logistic regression was used as the primary multivariable analysis. The proportional-odds assumption for the ordinal logistic regression model was evaluated using the Test of Parallel Lines. A non-significant result was considered evidence that the assumption was satisfied. Multiple linear regression using the same ordered numerical coding was performed to examine the consistency of the findings. Regression coefficients (B), standardized coefficients (β), 95% confidence intervals (CI), and *p*-values were reported. Statistical significance was established at a two-sided *p*-value < 0.05.

## 3. Results

### 3.1. Sociodemographic Characteristics of the Participants

A total of 323 patients were included in the study. The mean age of the participants was 30.92 ± 9.91 years. Females accounted for a larger proportion of the study population than males (195, 60.4% vs. 128, 39.6%). Most participants had completed a college education (252, 78.0%), followed by postgraduate education (37, 11.5%), whereas only a small proportion had primary (13, 4.0%) or high school education (21, 6.5%). Regarding monthly income, more than half of the participants (168, 52.0%) reported earning over 8000 RMB per month, while 84 (26.0%) earned between 5000 and 8000 RMB, 52 (16.1%) earned between 3000 and 5000 RMB, and 19 (5.9%) reported a monthly income below 3000 RMB. Detailed participant characteristics are presented in Table 1.

### 3.2. OHL

Overall, participants demonstrated relatively high levels of OHL (Table S1). Most respondents agreed or strongly agreed that they were able to understand written oral health materials (94.7%), identify reliable sources of oral health information (95.3%), communicate confidently with dentists (93.5%), and use oral health information to make informed decisions (81.7%). Similarly, a high proportion reported understanding the importance of preventive oral care (64.7%) and following dental instructions provided by dentists (66.3%). In contrast, fewer participants expressed confidence in evaluating the trustworthiness of oral health information, with approximately half agreeing or strongly agreeing with this statement (55.7%), while 44.3% selected a neutral response.

### 3.3. Oral Health Knowledge

Participants generally demonstrated good oral health knowledge (Table S2). More than 85% of respondents correctly identified that bleeding gums may indicate disease (86.7%), sugary food consumption increases the risk of dental caries (87.6%), and brushing teeth twice daily is recommended (89.8%). In contrast, only 54.2% correctly recognized that fluoride helps prevent tooth decay, making it the item with the lowest correct response rate.

### 3.4. Risk Behaviors

The distribution of risk behaviors is presented in Table S3. Frequent consumption of sweet foods and sugary beverages was common, with 41.2% of participants reporting daily intake for both behaviors. More than half of the participants (52.0%) reported skipping toothbrushing at night on a daily basis, while 63.5% indicated that they routinely delayed seeking dental treatment. In contrast, daily consumption of snacks before bedtime was relatively uncommon (4.3%). Current smoking and alcohol consumption were reported by 33.7% and 28.8% of participants, respectively.

### 3.5. Preventive Oral-Health Practices

The distribution of preventive oral-health practices is shown in Table S4. Most participants reported brushing their teeth at least twice daily (64.7%), using fluoride toothpaste (80.2%), attending regular dental check-ups (65.0%), and receiving professional dental cleaning within the previous year (74.9%). In contrast, regular use of dental floss (27.9%) and preventive dental visits (40.2%) were less commonly reported, while fewer than half of the participants indicated regular use of mouthwash (41.8%).

### 3.6. Self-Rated Oral Health Status

The self-rated oral health status of the participants is presented in Table 2. Nearly half of the participants rated their oral health as fair (47.1%), while 30.7% considered it good, and only 4.3% reported very good oral health. Dental pain was reported by 64.7% of participants, whereas gingival bleeding during toothbrushing was reported by 32.2%. Regarding overall satisfaction with oral health, the largest proportion of participants reported being dissatisfied (37.5%) or neutral (35.3%), while only 13.0% indicated that they were satisfied with their oral health.

### 3.7. Internal Consistency Reliability of the Questionnaire Domains

The internal consistency reliability of the questionnaire domains is presented in Table 3. The OHL and Oral Health Knowledge domains demonstrated acceptable internal consistency, with Cronbach’s α values of 0.775 and 0.701, respectively. The Risk Behaviors domain demonstrated excellent internal consistency reliability (Cronbach’s α = 0.925). The preventive oral-health practice items produced a Cronbach’s alpha coefficient of 0.926. However, because these items represent multiple distinct preventive behaviors, the resulting score should be interpreted as a preventive-practice index summarizing engagement in preventive oral-health practices rather than as a unidimensional psychometric scale.

### 3.8. Composite Questionnaire Scores

The mean OHL score was 31.16 ± 3.50 (observed range: 23–40), indicating generally favorable levels of OHL among the participants. The mean Oral Health Knowledge score was 3.18 ± 1.09 (range: 0–4). The mean Risk Behavior score was 6.22 ± 3.98 (range: 0–13), while the mean Preventive Oral-Health Practice score was 4.29 ± 2.64 (range: 0–7). Detailed descriptive statistics for the composite questionnaire scores are presented in Table 4.

### 3.9. Ordinal Logistic Regression Analysis

Ordinal logistic regression was performed to identify factors associated with self-rated oral health (Table 5). The model was statistically significant (χ^2^ = 29.793, *p* = 0.002) and demonstrated acceptable goodness-of-fit (Pearson *p* = 0.652). The pseudo-R^2^ values were Cox and Snell R^2^ = 0.088, Nagelkerke R^2^ = 0.098, and McFadden R^2^ = 0.040. The Test of Parallel Lines was non-significant (χ^2^ = 32.130, df = 22, *p* = 0.075), indicating that the proportional-odds assumption was satisfied. The analysis showed that higher risk behavior scores were significantly associated with lower odds of reporting better self-rated oral health (Estimate = −0.104, 95% CI: −0.161 to −0.047, *p* < 0.001). OHL, oral health knowledge, age, sex, and income were not significantly associated with self-rated oral health. Because higher self-rated oral health categories represented better perceived oral health, the negative ordinal logistic regression coefficient indicates lower odds of reporting better self-rated oral health as risk behavior scores increased.

### 3.10. Multiple Linear Regression and Spearman’s Rank Correlation Analysis

Multiple linear regression analysis was conducted to identify factors associated with risk behavior scores (Table 6). The overall model was statistically significant (F = 5.089, *p* < 0.001), explaining 14.0% of the variance in risk behavior scores (R^2^ = 0.140; adjusted R^2^ = 0.113). Age (β = 0.180, *p* = 0.001) and higher oral health knowledge scores (β = 0.199, *p* = 0.002) were positively associated with risk behavior scores, whereas higher OHL scores were negatively associated with risk behavior scores (β = −0.355, *p* < 0.001). Sex, education, and income were not significantly associated with risk behavior scores after adjustment. The tolerance values for OHL and oral health knowledge were 0.696 and 0.703, respectively, while the corresponding variance inflation factor (VIF) values were 1.437 and 1.423. These values indicate that problematic multicollinearity was not present and that the regression estimates were stable.

Multiple linear regression analysis was performed to identify factors associated with self-rated oral health (Table 7). The overall regression model was statistically significant (F = 2.862, *p* = 0.001), explaining 9.2% of the variance in self-rated oral health (R^2^ = 0.092; adjusted R^2^ = 0.060). Risk behavior scores were independently associated with poorer self-rated oral health (β = −0.214, *p* < 0.001). In addition, participants with college-level education reported significantly poorer self-rated oral health than those with postgraduate education (β = −0.187, *p* = 0.011). Age, sex, income, OHL, and oral health knowledge were not significantly associated with self-rated oral health after adjustment. Higher risk behavior scores represented greater engagement in unfavorable oral health-related behaviors, whereas higher self-rated oral health scores represented better perceived oral health. Therefore, the negative regression coefficient indicates that greater engagement in risk behaviors was associated with poorer self-rated oral health.

Table 8 presents Spearman’s rank correlation analysis among the main study variables. OHL was moderately and positively correlated with oral health knowledge (ρ = 0.602, *p* < 0.001). Age demonstrated weak positive correlations with both OHL (ρ = 0.161, *p* = 0.004) and oral health knowledge (ρ = 0.110, *p* = 0.048). A weak positive correlation was also observed between age and risk behaviors (ρ = 0.134, *p* = 0.016). OHL showed a weak negative correlation with risk behaviors (ρ = −0.238, *p* < 0.001), indicating that participants with higher levels of OHL tended to report fewer oral health-related risk behaviors. No statistically significant correlations were observed between preventive oral-health practices and age, OHL, oral health knowledge, or risk behaviors (all *p* > 0.05).

## 4. Discussion

This study investigated OHL, oral health knowledge, risk behaviors, preventive oral-health practices, and self-rated oral health among Chinese dental patients attending a tertiary hospital in Guangzhou. The findings partially supported the study hypotheses. Higher OHL was associated with greater oral health knowledge and lower levels of oral health-related risk behaviors, while greater engagement in risk behaviors was associated with poorer self-rated oral health. However, the relationship between oral health knowledge and behavioral outcomes appeared more complex than initially expected. Taken together, these findings suggest that OHL, oral health knowledge, and oral health-related behaviors are interrelated factors associated with self-rated oral health among dental patients [9,20,21].

The relatively high levels of OHL observed in this study are likely related to the characteristics of the study population. Participants were recruited from a tertiary-care dental hospital and were actively seeking dental services. Consequently, they may have had greater exposure to oral health information and more frequent interactions with dental professionals than the general population. Previous studies have reported that individuals with higher OHL are more likely to seek preventive dental care, understand health-related information, and communicate effectively with healthcare providers [15,22,23]. Participants also demonstrated generally good oral health knowledge, particularly regarding the relationship between sugary foods and dental caries, the significance of gingival bleeding, and recommended toothbrushing frequency. However, knowledge regarding the preventive benefits of fluoride was comparatively lower than other knowledge factors, suggesting an ongoing need for public education concerning evidence-based caries prevention strategies [24,25].

A major finding of the present study was the significant positive association between OHL and oral health knowledge. Participants with higher OHL scores tended to demonstrate greater oral health knowledge, which is consistent with previous evidence suggesting that health literacy facilitates the acquisition, understanding, evaluation, and application of health-related information [5,11,15]. Consequently, individuals with higher OHL may be better positioned to acquire accurate information regarding disease prevention and oral self-care practices. The positive correlation observed in this study further supports the conceptual distinction between literacy and knowledge while demonstrating their close interrelationship. Although OHL and oral health knowledge were correlated, collinearity diagnostics indicated no evidence of problematic multicollinearity (VIFs < 2.0). This finding suggests that the two variables captured related but distinct dimensions of oral health competence and that their simultaneous inclusion in the regression model did not compromise the stability of the estimates.

Importantly, OHL was negatively associated with oral health-related risk behaviors in both the correlation and multivariable regression analyses. Participants with higher OHL scores reported fewer unfavorable behaviors that may increase their risk of oral diseases. This finding is consistent with previous studies showing that higher OHL is associated with improved oral hygiene practices, healthier lifestyles, greater utilization of preventive dental services, and better oral health outcomes [25,26,27,28]. Individuals with adequate OHL may be more capable of understanding risk-related information, evaluating the credibility of health messages, and translating oral health recommendations into daily behavioral practices [29]. Therefore, OHL may be associated with healthier decision-making and behavioral choices beyond simple knowledge acquisition. These findings support the relevance of OHL to oral health behavior and suggest that oral health promotion initiatives should incorporate literacy-enhancing interventions as part of comprehensive prevention strategies.

Interestingly, oral health knowledge demonstrated a weaker relationship with behavioral outcomes than OHL. Although oral health knowledge was correlated with OHL, it was not significantly associated with risk behaviors in the bivariate analysis, suggesting that knowledge alone may be insufficient to produce consistent behavioral change. This finding may be interpreted within established behavioral science frameworks, including the Health Belief Model and the Theory of Planned Behavior, which propose that health-related behaviors are influenced not only by knowledge but also by perceived susceptibility, perceived benefits and barriers, self-efficacy, social norms, and behavioral intentions [30]. Individuals may therefore possess adequate knowledge regarding oral disease prevention while continuing to engage in unfavorable behaviors because of habitual practices, competing priorities, limited motivation, or environmental constraints. Consequently, educational strategies that focus on knowledge transmission may have limited effectiveness unless accompanied by interventions that strengthen motivation, self-efficacy, and behavioral skills [31,32]. Moreover, oral health knowledge was positively associated with risk behavior scores in the adjusted regression analysis. This finding should be interpreted cautiously and does not indicate that greater oral health knowledge causes more risk behaviors. One possible explanation is the well-documented “knowledge–behavior gap” whereby greater knowledge does not necessarily translate into healthier behaviors. In the present study, participants were actively receiving dental care in a tertiary hospital setting and may therefore have been repeatedly exposed to oral health education and counseling. Consequently, some individuals who engage in unfavorable oral health-related behaviors may have acquired greater knowledge through previous dental consultations while continuing to maintain established behavioral patterns. In addition, health-related behaviors are influenced by a range of factors beyond knowledge, including habits, motivation, self-efficacy, social influences, and environmental constraints [5,15,33]. Therefore, individuals may possess adequate knowledge regarding recommended oral health practices while continuing to engage in unfavorable behaviors. These findings support the growing view that information acquisition alone may be insufficient to achieve sustained behavioral change and that behavior-focused interventions may also be required.

The finding that OHL showed a stronger association with behavioral outcomes than oral health knowledge is particularly noteworthy [34]. Although knowledge provides individuals with information regarding oral disease prevention, literacy encompasses broader competencies, including the ability to obtain, evaluate, interpret, and apply health information in real-world situations. This distinction may help explain why literacy demonstrated a stronger relationship with oral health-related behaviors than knowledge alone. From a behavioral science perspective, acquiring information does not necessarily result in behavioral change because health-related decisions are also influenced by motivation, self-efficacy, social norms, and environmental factors. These findings support the growing view that oral health promotion programs should move beyond information delivery and incorporate strategies that facilitate the practical application of health knowledge in daily life [32]. The observed association between oral health-related risk behaviors and poorer self-rated oral health underscores the importance of behavioral factors in shaping individuals’ perceptions of their oral health status. While OHL and oral health knowledge were associated with behavioral outcomes, the present study was not designed to determine whether oral health-related behaviors mediate the relationship between these factors and oral health outcomes. This finding is consistent with previous studies reporting that self-rated oral health reflects not only clinical oral conditions but also the cumulative contribution of oral hygiene practices, dietary habits, smoking behavior, and patterns of dental service utilization [35,36,37]. The results suggest that oral health promotion strategies may achieve greater impact when they focus not only on improving knowledge and literacy but also on facilitating the adoption and maintenance of healthier behaviors. Therefore, interventions designed to reduce oral health-related risk behaviors may represent an important factor associated with perceived oral health among dental patients.

The regression models explained 14.0% of the variance in risk behavior scores and 9.2% of the variance in self-rated oral health. These findings indicate that OHL, oral health knowledge, risk behaviors, and selected sociodemographic characteristics were significantly associated with the outcomes examined. Oral health-related behaviors and self-rated oral health are complex and influenced by multiple determinants beyond those assessed in the present study [11,29,32]. From a practical perspective, the results suggest that OHL and oral health knowledge represent only part of a broader set of factors influencing oral health-related behaviors and perceptions [11,31,32]. Other psychological, social, behavioral, clinical, and environmental factors not measured in the present study may also contribute to the observed outcomes [31,32,34]. Therefore, interventions aimed at improving oral health literacy and knowledge may represent an important component of oral health promotion but are unlikely to fully explain or modify oral health-related behaviors and outcomes on their own [11,29,31,32].

Several limitations should be acknowledged. First, the cross-sectional design precludes causal inferences regarding the relationships among OHL, oral health knowledge, behaviors, and oral health outcomes. Second, all study variables, including OHL, oral health knowledge, oral health-related behaviors, and self-rated oral health, were assessed using self-rated questionnaires. Consequently, the findings may be affected by recall bias and social desirability bias. Because data collection was conducted within a dental hospital setting, participants may have been more likely to report behaviors and abilities that were perceived as socially acceptable or consistent with recommended oral health practices. As a result, favorable behaviors and literacy-related competencies may have been overestimated, whereas unfavorable behaviors may have been underreported. Future studies should consider incorporating objective assessments of OHL, clinical oral health status, and oral health-related behaviors to complement self-rated measures. Third, participants were recruited from a single tertiary hospital using convenience sampling. Individuals seeking care at tertiary referral centers may have greater exposure to dental services, preventive advice, and oral health information than the general population. Therefore, the relatively high levels of OHL and oral health knowledge observed in this study may partially reflect characteristics of the study population and may not be representative of broader community populations. Fourth, although the questionnaire was adapted from previously published studies and underwent expert review and pilot testing, additional psychometric evaluations such as exploratory factor analysis, confirmatory factor analysis, and test–retest reliability assessment were not performed. Additionally, oral health knowledge was assessed using only four items and may not fully capture its multidimensional nature. Future studies should consider using more comprehensive knowledge instruments to improve content validity and provide a broader assessment of oral health-related knowledge. Finally, unmeasured psychological, behavioral, clinical, and environmental factors may have influenced the observed associations. Future multicenter studies using more representative sampling approaches and longitudinal designs are warranted to improve external validity and to better elucidate the pathways linking OHL, oral health knowledge, behaviors, and oral health outcomes.

## 5. Conclusions

This study demonstrated that Chinese dental patients attending a tertiary hospital generally possessed relatively high levels of OHL and oral health knowledge. OHL was positively associated with oral health knowledge and negatively associated with oral health-related risk behaviors. Furthermore, higher OHL was independently associated with lower risk behavior scores, while greater engagement in risk behaviors was associated with poorer self-rated oral health. Together, these findings suggest that OHL, oral health knowledge, and oral health-related behaviors are closely interrelated among Chinese dental patients and may represent important considerations in oral health promotion. Future studies are needed to clarify the temporal relationships among these factors and to determine how they may be incorporated into effective oral health promotion strategies.

## Figures and Tables

**Table 1:** Sociodemographic Characteristics of Participants (*n* = 323).

Variable	Category	*n* (%)
Age (18–62 years)	Mean ± SD	30.92 ± 9.91
Sex	Male	128 (39.6)
	Female	195 (60.4)
Education	Primary or below	13 (4.0)
	Secondary	21 (6.5)
	College	252 (78.0)
	Postgraduate	37 (11.5)
Income	<3000	19 (5.9)
	3000–5000	52 (16.1)
	5000–8000	84 (26.0)
	>8000	168 (52)

**Table 2:** Self-Rated Oral Health Status of Participants.

Variable	Response	*n* (%)
Self-rated oral health	Poor	58 (18.0)
	Fair	152 (47.1)
	Good	99 (30.7)
	Very good	14 (4.3)
	Excellent	0 (0)
Dental pain	No	114 (35.3)
	Yes	209 (64.7)
Gingival bleeding during toothbrushing	No	219 (67.8)
	Yes	104 (32.2)
Satisfaction with oral health	Very dissatisfied	46 (14.2)
	Dissatisfied	121 (37.5)
	Neutral	114 (35.3)
	Satisfied	42 (13.0)

**Table 3:** Internal Consistency Reliability of the Questionnaire Domains.

Domain	No. of Items	Cronbach’s α	Interpretation
OHL	8	0.775	Acceptable
Oral Health Knowledge	4	0.701	Acceptable
Risk Behaviors	7	0.925	Excellent
Preventive Oral-Health Practice Index	7	0.926	Reported for descriptive purposes *

* The preventive oral-health practice index summarizes multiple distinct preventive behaviors and should not be interpreted as a unidimensional psychometric construct. Cronbach’s alpha is reported for descriptive purposes only. Abbreviations: OHL: oral health literacy.

**Table 4:** Descriptive Statistics of the Composite Questionnaire Scores.

Domain	Possible Score Range	Mean ± SD	Observed Range
OHL	8–40	31.16 ± 3.50	23–40
Oral Health Knowledge	0–4	3.18 ± 1.09	0–4
Risk Behaviors	0–14	6.22 ± 3.98	0–13
Preventive Oral-Health Practice Index	0–7	4.29 ± 2.64	0–7

Abbreviations: OHL: oral health literacy; SD: standard deviation.

**Table 5:** Ordinal Logistic Regression Analysis Predicting Self-Rated Oral Health.

Predictor	Estimate	SE	Wald χ^2^	*p*-Value	95% CI
Age	0.007	0.011	0.360	0.549	−0.015 to 0.029
Sex (Male vs. Female)	0.409	0.221	3.441	0.064	−0.023 to 0.841
Education (Primary)	−0.426	0.631	0.456	0.500	−1.663 to 0.811
Education (Secondary)	0.043	0.535	0.006	0.936	−1.006 to 1.092
Education (College)	−0.796	0.346	5.275	0.022	−1.475 to −0.117
Income (<3000 RMB)	−0.730	0.481	2.302	0.129	−1.674 to 0.213
Income (3000–5000 RMB)	0.263	0.305	0.746	0.388	−0.334 to 0.861
Income (5000–8000 RMB)	0.467	0.259	3.237	0.072	−0.042 to 0.975
OHL	0.002	0.038	0.003	0.954	−0.072 to 0.076
Oral Health Knowledge	−0.058	0.117	0.247	0.619	−0.287 to 0.171
Risk Behaviors	−0.104	0.029	12.808	<0.001	−0.161 to −0.047

Model statistics: Ordinal logistic regression model χ^2^ = 29.793, df = 11, *p* = 0.002. Pearson goodness-of-fit χ^2^ = 901.694, df = 919, *p* = 0.652. Deviance goodness-of-fit χ^2^ = 703.937, df = 919, *p* = 1.000. Cox and Snell R^2^ = 0.088, Nagelkerke R^2^ = 0.098, McFadden R^2^ = 0.040. Test of Parallel Lines: χ^2^ = 32.130, df = 22, *p* = 0.075. Abbreviations: OHL: oral health literacy; SE: Standard Error; CI: Confidence Interval; Wald χ^2^: Wald chi-square statistic.

**Table 6:** Multiple Linear Regression Analysis Predicting Risk Behaviors Among Chinese Dental Patients.

Predictor	B	SE	Standardized β	t	*p*-Value	95% CI
Constant	14.773	2.126	—	6.948	<0.001	10.589 to 18.956
Age	0.072	0.022	0.180	3.302	0.001	0.029 to 0.115
Male (vs. Female)	0.434	0.435	0.053	0.996	0.320	−0.423 to 1.291
Primary Education (vs. Postgraduate)	2.053	1.247	0.102	1.646	0.101	−0.401 to 4.508
Secondary Education (vs. Postgraduate)	−0.409	1.061	−0.025	−0.386	0.700	−2.498 to 1.679
College Education (vs. Postgraduate)	−0.607	0.681	−0.063	−0.891	0.374	−1.948 to 0.734
Income < 3000 RMB (vs. >8000 RMB)	−1.472	0.946	−0.087	−1.556	0.121	−3.334 to 0.390
Income 3000–5000 RMB (vs. >8000 RMB)	−0.919	0.604	−0.085	−1.522	0.129	−2.106 to 0.269
Income 5000–8000 RMB (vs. >8000 RMB)	−0.183	0.513	−0.020	−0.356	0.722	−1.192 to 0.827
OHL	−0.403	0.071	−0.355	−5.641	<0.001	−0.544 to −0.263
Oral Health Knowledge	0.725	0.228	0.199	3.177	0.002	0.276 to 1.174

Model statistics: R = 0.374; R^2^ = 0.140; Adjusted R^2^ = 0.113; F(10,312) = 5.089; *p* < 0.001; Abbreviations: OHL: oral health literacy; SE: Standard Error; CI: Confidence Interval.

**Table 7:** Multiple Linear Regression Analysis Predicting Self-Rated Oral Health Among Chinese Dental Patients.

Predictor	B	SE	Standardized β	t	*p*-Value	95% CI
Constant	2.601	0.464	—	5.609	<0.001	1.689 to 3.513
Age	0.002	0.005	0.023	0.412	0.681	−0.007 to 0.011
Male (vs. Female)	0.160	0.089	0.100	1.806	0.072	−0.014 to 0.334
Primary Education (vs. Postgraduate)	−0.212	0.254	−0.053	−0.835	0.405	−0.713 to 0.288
Secondary Education (vs. Postgraduate)	−0.001	0.216	0.000	−0.003	0.998	−0.425 to 0.423
College Education (vs. Postgraduate)	−0.353	0.138	−0.187	−2.551	0.011	−0.626 to −0.081
Income < 3000 RMB (vs. >8000 RMB)	−0.317	0.193	−0.095	−1.646	0.101	−0.697 to 0.062
Income 3000–5000 RMB (vs. >8000 RMB)	0.099	0.123	0.047	0.806	0.421	−0.143 to 0.341
Income 5000–8000 RMB (vs. >8000 RMB)	0.180	0.104	0.101	1.730	0.085	−0.025 to 0.385
OHL	0.001	0.015	0.005	0.072	0.943	−0.029 to 0.031
Oral Health Knowledge	−0.013	0.047	−0.018	−0.270	0.788	−0.105 to 0.080
Risk Behaviors	−0.042	0.011	−0.214	−3.668	<0.001	−0.065 to −0.020

Model statistics: R = 0.303; R^2^ = 0.092; Adjusted R^2^ = 0.060; F(11,311) = 2.862; *p* = 0.001. Abbreviations: OHL: oral health literacy; SE: Standard Error; CI: Confidence Interval.

**Table 8:** Spearman’s Rank Correlation Matrix Among the Main Study Variables.

Variable	1	2	3	4	5
Age	1.000				
Preventive oral-health practices	0.029	1.000			
OHL	0.161 **	−0.040	1.000		
Oral health knowledge	0.110 *	−0.036	0.602 **	1.000	
Risk behaviors	0.134 *	−0.070	−0.238 **	−0.013	1.000

Abbreviations: OHL: oral health literacy; * *p* < 0.05; ** *p* < 0.01.

## Data Availability

The original contributions presented in this study are included in the article. Further inquiries can be directed to the corresponding author.

## Supplementary Materials

The following supporting information can be downloaded at: https://www.mdpi.com/article/10.3390/healthcare14203444/s1, File S1 Study Questionnaire; Figure S1. Participant recruitment and selection flowchart. A total of 343 eligible patients were invited to participate. Of the initial 323 participants, 7 questionnaires were incomplete and were replaced by 7 participants from the 20 additional participants invited for this purpose. The remaining 13 additional participants were not included in the final analysis. A total of 323 participants with complete questionnaires were included in the final analysis; Table S1. Distribution of Responses for Oral Health Literacy; Table S2. Distribution of Responses for Oral Health Knowledge; Table S3. Distribution of Responses for Risk Behavior; Table S4. Distribution of Responses for Preventive Oral-Health Practices.

## References

[1] Peres M.A., Macpherson L.M.D., Weyant R.J., Daly B., Venturelli R., Mathur M.R., Listl S., Celeste R.K., Guarnizo-Herreño C.C., Kearns C. (2019). Oral diseases: A global public health challenge. Lancet.

[2] World Health Organization Oral Health. https://www.who.int/health-topics/oral-health.

[3] Denis F., Clement C. (2025). Oral Health: A Major Global Public Health Concern. J. Clin. Med..

[4] Das H. (2026). Why prevention is failing in oral health and a wake-up call for rethinking strategies for the world’s most widespread non-communicable disease. Discov. Public Health.

[5] Firmino R.T., Ferreira F.M., Paiva S.M., Granville-Garcia A.F., Fraiz F.C., Martins C.C. (2017). Oral health literacy and associated oral conditions: A systematic review. J. Am. Dent. Assoc..

[6] Meyer-Hofmann A.M., Barbe A.G., von Kohout M., Aarabi G., Deeg I., Wicht M.J., Derman S.H.M. (2026). Assessment of oral health literacy and influencing factors: Retrospective analysis of patients treated by dental students. Eur. J. Dent. Educ..

[7] Liu J., Zhang S.S., Zheng S.G., Xu T., Si Y. (2016). Oral health status and oral health care model in China. Chin. J. Dent. Res..

[8] Cao C., Liao S., Cao W., Guo Y., Hong Z., Ren B., Hu Z., Bai Z. (2023). Differences in the association of oral health knowledge, attitudes, and practices with frailty among community-dwelling older people in China. BMC Oral Health.

[9] Qin W., Liu N., Wang Q., Dong Y., Jiang L. (2024). Oral health literacy and patient education practices among non-dental professionals in Chongqing, China: A cross-sectional study. Med. Sci. Monit..

[10] Zhou X., Xu X., Li J., Hu D., Hu T., Yin W., Fan Y., Zhang X. (2018). Oral health in China: From vision to action. Int. J. Oral Sci..

[11] Yu S., Huang S., Song S., Lin J., Liu F. (2024). Impact of oral health literacy on oral health behaviors and outcomes among older adults: A scoping review. BMC Geriatr..

[12] Jiang R., Yu J., Islam R., Li X., Nie E. (2023). Dental caries prevention knowledge, attitudes, and practice among patients at a university hospital in Guangzhou, China. Medicina.

[13] Nie E., Jiang R., Islam R., Li X., Yu J. (2024). Evaluation of caries risk assessment practices among dental practitioners in Guangzhou, China: A cross-sectional study. Front. Oral Health.

[14] Xiao F., Yu D., Huang Y., Zhang T., He H., Luo W., Ye L., Qin W. (2026). Oral health knowledge, attitudes, and practices and associated factors among non-dental healthcare professionals in South China: A cross-sectional study. Front. Public Health.

[15] Firmino R.T., Martins C.C., Faria L.D.S., Martins Paiva S., Granville-Garcia A.F., Fraiz F.C., Ferreira F.M. (2018). Association of oral health literacy with oral health behaviors, perception, knowledge, and dental treatment-related outcomes: A systematic review and meta-analysis. J. Public Health Dent..

[16] King S., Thaliph A., Laranjo L., Smith B.J., Eberhard J. (2023). Oral health literacy, knowledge and perceptions in a socially and culturally diverse population: A mixed methods study. BMC Public Health.

[17] Wang W., Xiao C., Li S., Li M., Zheng F., Shang L., Li R. (2025). Oral health literacy and behaviors among Chinese university students: A comparative study of medical and non-medical students. BMC Oral Health.

[18] Flynn P., Ingleshwar A., Chen X., Feuerstahler L., Reibel Y., John M.T. (2023). Validation of the HeLD-14 functional oral health literacy instrument in a general population. PeerJ.

[19] Morita I., Nakagaki H., Toyama A., Hayashi M., Shimozato M., Watanabe T., Tohmatsu S., Igo J., Sheiham A. (2006). Behavioral factors to include in guidelines for lifelong oral healthiness: An observational study in Japanese adults. BMC Oral Health.

[20] An R., Li S., Li Q., Luo Y., Wu Z., Liu M., Chen W. (2022). Oral health behaviors and oral health-related quality of life among dental patients in China: A cross-sectional study. Patient Prefer. Adherence.

[21] Adunola F., Macek M.D., Atchison K., Akinkugbe A. (2023). Association between oral health knowledge, perceived oral health-related quality of life, perceived oral health status and emergency department and/or urgent care visit: Results from the multi-site oral health literacy study. J. Public Health Dent..

[22] Silva-Junior M.F., Rosário de Sousa M.D.L., Batista M.J. (2021). Health literacy on oral health practice and condition in an adult and elderly population. Health Promot. Int..

[23] Ueno M., Takeuchi S., Oshiro A., Kawaguchi Y. (2013). Relationship between oral health literacy and oral health behaviors and clinical status in Japanese adults. J. Dent. Sci..

[24] An R., Chen W.F., Li S., Wu Z., Liu M., Sohaib M. (2022). Assessment of oral health literacy and oral health behaviors among nurses in China: A cross-sectional study. BMC Oral Health.

[25] Yeh C.-H., Wang Y.-L., Vo T.T.T., Lee Y.-C., Lee I.-T. (2025). Fluoride in Dental Caries Prevention and Treatment: Mechanisms, Clinical Evidence, and Public Health Perspectives. Healthcare.

[26] Lee J.Y., Divaris K., Baker A.D., Rozier R.G., Lee S.Y., Vann W.F. (2011). Oral health literacy levels among a low-income WIC population. J. Public Health Dent..

[27] Blizniuk A., Ueno M., Zaitsu T., Kawaguchi Y. (2015). Association of oral health literacy with oral health behaviour and oral health status in Belarus. Community Dent. Health.

[28] Kandasamy G., Almaghaslah D., Vasudevan R., Shorog E., Alshahrani A.M., Alsawaq E., Alzlaiq W., Prabahar K., Veeramani V.P., Alshareef H. (2023). Assessment of oral health literacy and oral health-related quality of life in Saudi university students: A cross-sectional study. J. Oral Rehabil..

[29] Batista M.J., Lawrence H.P., Sousa M.D.L.R. (2017). Oral health literacy and oral health outcomes in an adult population in Brazil. BMC Public Health.

[30] Alamer A.S. (2024). Behavior Change Theories and Models Within Health Belief Model Research: A Five-Decade Holistic Bibliometric Analysis. Cureus.

[31] Sheeran P., Maki A., Montanaro E., Avishai-Yitshak A., Bryan A., Klein W.M., Miles E., Rothman A.J. (2016). The impact of changing attitudes, norms, and self-efficacy on health-related intentions and behavior: A meta-analysis. Health Psychol..

[32] Thirasupa N., Kasevayuth K., Intarakamhang U. (2025). Behavioral theories and health literacy influencing oral health behaviors: A systematic review. J. Behav. Sci..

[33] Schroeder K.L., McLeod C., Clester S., Cheung H.J., Heaton L.J., Tranby E.P. (2025). Oral health literacy from a person-centered focus in the United States: A narrative review. BMC Oral Health.

[34] Baxter M.L. (2026). Systematic Review and Analysis of Measures of Oral Health Literacy. J. Dent. Educ..

[35] Vu C.T.B., Pham T.A.V. (2026). Association between Oral Health Perception, Dental Knowledge, and Habits and Oral Health Status among Students at Vietnam National University, Ho Chi Minh City: A Cross-Sectional Study. J. Int. Soc. Prev. Community Dent..

[36] Tadin A., Badrov M. (2024). Oral Health Knowledge, Self-Assessed Oral Health Behavior, and Oral Hygiene Practices among the Adult General Population in Croatia. Healthcare.

[37] Helmi M. (2025). Impacts of oral health on life quality metrics: Correlations with job function, psychological well-being, self-perception, and dietary behavior. Front. Oral Health.

