# Supplementary material for: Association of Oral Health Literacy and Oral Health Knowledge with Risk Behaviors and Self-Rated Oral Health Among Chinese Dental Patients: A Hospital-Based Cross-Sectional Study in Guangzhou
Source: Healthcare (Basel). 2026 Oct 14;14(20):3444. doi: 10.3390/healthcare14203444 (PMC13704629; doi:10.3390/healthcare14203444)
Supplement: Supplementary file 1 [file healthcare-14-03444-s001.zip › File S1 Study Questionnaire.pdf]

Study Questionnaire

Section 1. Sociodemographic Characteristics

1. Age: _____ years

2. Sex: □ Male □ Female

3. Highest Educational Attainment: □ Primary school or below □ Secondary school □ College/University □ Postgraduate

4. Monthly Income (RMB): □ <3000 □ 3000–5000 □ 5000–8000 □ >8000

Section 2. Oral Health Literacy (OHL)

Please indicate your level of agreement with the following statements (Strongly Disagree / Disagree / Neutral / Agree / Strongly Agree).

1. I can understand oral health information provided by dentists.
2. I can understand written oral health materials.
3. I know where to find reliable oral health information.
4. I can judge whether oral health information is trustworthy.
5. I can follow dental instructions provided by dentists.
6. I feel confident discussing oral health issues with dentists.
7. I understand the importance of preventive oral care.
8. I can use oral health information to make decisions regarding my oral health.

Section 3. Oral Health Knowledge

Please indicate your level of agreement with the following statements (Incorrect/Correct)

1. Fluoride helps prevent tooth decay.
2. Bleeding gums may indicate oral disease.
3. Frequent consumption of sugary foods increases the risk of dental caries.
4. Teeth should be brushed at least twice daily.

Section 4. Oral Health-Related Risk Behaviors

Please indicate your level of agreement with the following statements (Never/Sometimes or Weekly/Daily)

1. How often do you consume sweet foods?
2. How often do you consume sugary beverages?
3. How often do you eat snacks before bedtime?

4. Smoking status: Never smoker / Former smoker / Current smoker
5. Alcohol consumption: Never / Weekly-Monthly
6. How often do you skip toothbrushing at night?
7. How often do you delay seeking dental treatment when needed?

Section 5. Preventive Oral-Health Practices

Please indicate your level of agreement with the following statements (Yes/No)

1. Do you brush your teeth at least twice daily?
2. Do you use fluoride toothpaste?
3. Do you regularly use dental floss?
4. Do you regularly use mouthwash?
5. Do you attend regular dental check-ups?
6. Have you received professional dental cleaning during the past year?
7. Do you attend preventive dental visits?

Section 6. Self-Rated Oral Health

1. How would you rate your overall oral health? (Poor / Fair / Good / Very Good / Excellent)
2. Have you experienced dental pain during the past 12 months?
3. Do your gums bleed during toothbrushing?
4. Overall, how satisfied are you with your oral health? (Very Dissatisfied / Dissatisfied / Neutral / Satisfied)
