# Supplementary material for: Association of Oral Health Literacy and Oral Health Knowledge with Risk Behaviors and Self-Rated Oral Health Among Chinese Dental Patients: A Hospital-Based Cross-Sectional Study in Guangzhou
Source: Healthcare (Basel). 2026 Oct 14;14(20):3444. doi: 10.3390/healthcare14203444 (PMC13704629; doi:10.3390/healthcare14203444)
Supplement: Supplementary file 1 [file healthcare-14-03444-s001.zip › Supplementary Materials Figure and Tables.pdf]

Supplementary Materials:

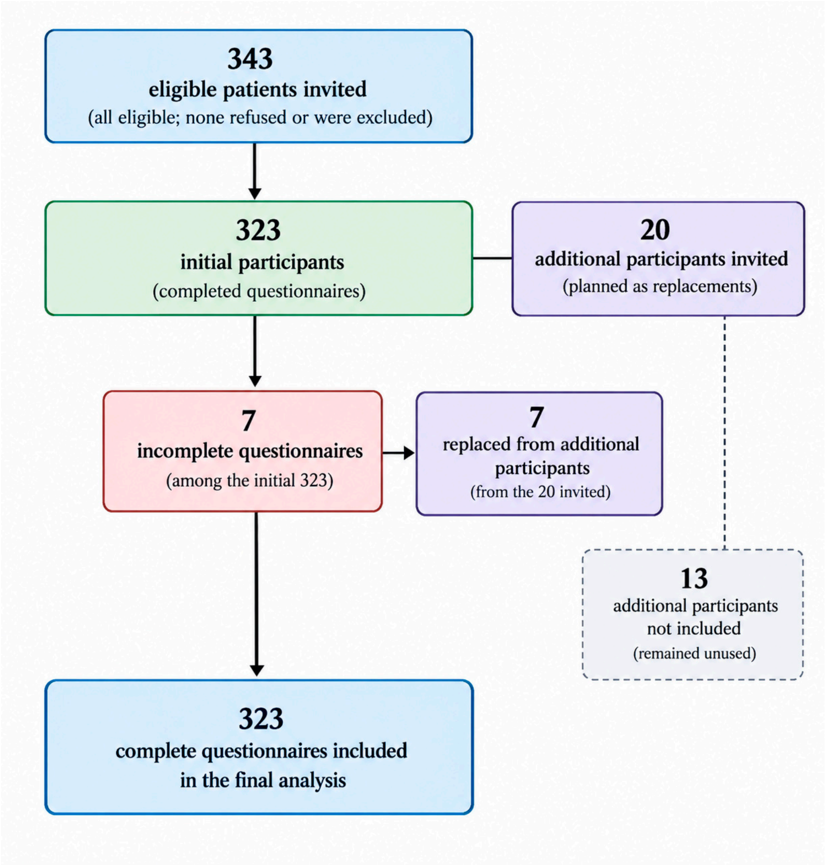

Figure S1. Participant recruitment and selection flowchart. A total of 343 eligible patients were invited to participate. Of the initial 323 participants, 7 questionnaires were incomplete and were replaced by 7 participants from the 20 additional participants invited for this purpose. The remaining 13 additional participants were not included in the final analysis. A total of 323 participants with complete questionnaires were included in the final analysis.

Table S1. Distribution of Responses for Oral Health Literacy.

Variable	Response	n (%)
**I can understand oral health information from dentists**	Disagree	1 (0.3)
	Neutral	84 (26.0)
	Agree	194 (60.1)
	Strongly agree	44 (13.6)
**I can understand written oral health materials**	Neutral	17 (5.3)
	Agree	242 (74.9)
	Strongly agree	64 (19.8)
**I know where to find reliable oral health information**	Neutral	15 (4.6)
	Agree	274 (84.8)
	Strongly agree	34 (10.5)
**I can judge whether oral health information is trustworthy**	Neutral	143 (44.3)
	Agree	157 (48.6)
	Strongly agree	23 (7.1)
**I can follow dental instructions**	Disagree	1 (0.3)
	Neutral	108 (33.4)
	Agree	183 (56.7)
	Strongly agree	31 (9.6)
**I feel confident discussing oral health with dentists**	Neutral	21 (6.5)
	Agree	244 (75.5)
	Strongly agree	58 (18.0)
**I understand the importance of preventive oral care**	Neutral	114 (35.3)
	Agree	189 (58.5)
	Strongly agree	20 (6.2)
**I can use oral health information to make decisions**	Neutral	59 (18.3)
	Agree	243 (75.2)
	Strongly agree	21 (6.5)

Note: All oral health literacy items were assessed using a five-point Likert scale (strongly disagree, disagree, neutral, agree, and strongly agree). Response categories with zero frequencies are not displayed in the table.

Table S2. Distribution of Responses for Oral Health Knowledge.

Variable	Response	n (%)
**Fluoride prevents tooth decay**	Incorrect	148 (45.8)
	Correct	175 (54.2)
**Bleeding gums indicate disease**	Incorrect	43 (13.3)
	Correct	280 (86.7)
**Sugary foods increase cavities**	Incorrect	40 (12.4)
	Correct	283 (87.6)
**Brushing twice daily is recommended**	Incorrect	33 (10.2)
	Correct	290 (89.8)

Table S3. Distribution of Responses for Risk Behavior.

Variable	Response	n (%)
**Sweet food consumption**	Never	12 (3.7)
	Sometimes/weekly	178 (55.1)
	Daily	133 (41.2)
**Sugary beverage consumption**	Never	12 (3.7)
	Sometimes/weekly	178 (55.1)
	Daily	133 (41.2)
**Snacking before bedtime**	Never	168 (52.0)
	Sometimes/weekly	141 (43.7)
	Daily	14 (4.3)
**Smoking**	Never	213 (65.9)
	Former	1 (0.3)
	Current	109 (33.7)
**Alcohol consumption**	Never	230 (71.2)
	Weekly/Monthly	93 (28.8)
**Skipping toothbrushing at night**	Never	144 (44.6)
	Sometimes	11 (3.4)
	Daily	168 (52.0)
**Delaying dental treatment**	Never	97 (30.0)
	Sometimes	21 (6.5)
	Always	205 (63.5)

Table S4. Distribution of Responses for Preventive Oral-Health Practices.

Variable	Response	n (%)
**Brushing teeth ≥2 times/day**	Yes	209 (64.7)
	No	114 (35.3)
**Use of fluoride toothpaste**	Yes	259 (80.2)
	No / Don't know	64 (19.8)
**Regular dental floss use**	Regular	90 (27.9)
	No/Rarely/Sometimes	233 (72.1)
**Regular mouthwash use**	Regular	135 (41.8)
	No/Rarely/Sometimes	188 (58.2)
**Regular dental check-up**	Yes	210 (65.0)
	No	113 (35.0)
**Professional dental cleaning during the previous year**	Yes	242 (74.9)
	No	81 (25.1)
**Preventive dental visit**	Yes	130 (40.2)
	No	193 (59.8)
